# Nitrous Oxid Oxygen Analgesia

**Published:** 1914-07-15

**Authors:** Moses Salzer

**Affiliations:** Cincinnati


### NITROUS OXID OXYGEN ANALGESIA.

BY MOSES SALZER, M. D., CINCINNATI.

From time immemorial various drugs have been used to produce a state of analgesia, by which is meant simply insensitiveness to pain, nothing more, nothing less, and is to be sharply distinguished from the state of anesthesia, in which consciousness is lost.

It is only recently, however, that nitrous oxid has been used to any extent for this purpose. Whether the use of this gas for analgesic purposes is a mere passing fancy or fad, the future alone can decide, but nevertheless the fact remains that this gas possesses many qualities which render it peculiarly fit for the production of analgesia.

In the first place, it is absolutely safe, even in the hands of the inexperienced, if the patient is not carried beyond the analgesic state.

Second, when administered with oxygen or a plentiful supply of air, it is not unpleasant to take; quite the reverse, it produces a sense of pleasing exhilaration.

Third, it is not followed by serious after-effects of any kind, no matter how long continued. The worst that could possibly happen is that the patient may become slightly nauseated or vomit.

Added to these factors of safety and pleasantness is the fact that nitrous oxid 's actually capable of eliminating pain without causing loss of consciousness. Its effects can be abolished or increased almost immediately at the will of the patient or administrator and contrary to any other analgesia-producing agent, its effects stop as soon as the administration ceases.

In other words, it is an almost ideal agent for the production of analgesia. And, let us not forget also that it does nothing more, either, than relieve pain when used for analgesic purposes and is not pushed to the state of general anesthesia. Nitrous oxid is not a general cure-all of the horrors of the dentist's chair.

The question now arises, why all this discussion, if nitrous oxid is capable of producing true analgesia and is absolutely safe? There can be no discussion of this point if we limit the use of this gas to the easing of pain alone. It is when we try to do more that we are doomed to failure. The sound of the bur, for instance, cannot be entirely eliminated, short of general anesthesia, and where this sensation is particularly unpleasant nitrous oxid analgesia will not always be a total success. The sense of touch, or the knowledge that something is being done, is also retained.

Nitrous oxid short of general anesthesia is not a success in "nervous" patients whose nervousness is not the result of the pain caused, or about to be caused, during the dental operation. I wish to report in this connection a case of Dr. R——'s. The patient was a highly-strung nervous boy of 14 who began to fidget and squirm even before he sat down in the chair. As a test, Dr. R—— placed the bur against the side of a perfectly sound tooth and the boy let out a most unearthly yell. Three cavities, however, were prepared for this boy and he reluctantly admitted after it was all over that it really did not hurt so much after all and that he would prefer to have his work done under gas in the future.

It is not a question of pain or no pain in these cases and it is utterly ridiculous, under the circumstances, to rely solely on a pain-relieving measure when what is needed is something to quiet the nerves.

We have the same condition to meet in general surgery. There are many, many operations that can be and ought to be done under local anesthesia or analgesia, but the patients are too nervous and do not want to be conscious of what is going on. The very idea and not the pain gives them the "fidgets" and prevents skillful and successful work. It is possible, however, in this class of cases, after they have once experienced real painless dentistry under nitrous oxid, that they may return a second time more composed and better subjects for analgesia. In other words, they must be educated up to it.

As a class, these patients will do better by being given some preliminary medication, such as morphia or bromides.

In suitable cases, however, i. e., cases where the pain caused by the dental manipulation itself is the cause of the patient's nervousness, it is truly marvelous what can be accomplished without causing the slightest discomfort to the patient. In one case Dr. R—— prepared eleven cavities in an hour and a quarter, any one of which, he declared, would have taken almost an hour in this patient, who was extremely sensitive to pain and to the heat of the bur which can be totally disregarded under analgesia. After it was all over, the patient declared that he would not mind it for another hour and that he had had a very pleasant time. Dr. R—— was able to extract a pulp without causing the patient any pain and without loss of consciousness.

Nitrous oxid acts by producing an intoxication controlled at the will of the operator. The patient may be made just happy drunk or dead drunk, just as may be desired. Fortunately they very rarely become crazy or fighting drunk.

Among the disadvantages, the question of expense must be considered. The cost of the gas and oxygen is about three dollars an hour and, added to this, will be the expense of having some one to administer it. From what little experience I have had I do not see how it would be possible for the dentist to operate and watch his patient at the same time. It is true that there are various devices on the market by which the patient may administer his own anesthetic but these are of necessity incapable of producing a smooth analgesia, for the patient's hand will not drop before he really is anesthetized; and secondly, instead of the arm relaxing, there may be a tonic contraction of the muscles of the arm which will hold the mask tighter than ever to the nose. By practice, however, a certain measure of regular analgesia can be produced by these devices. The question of the necessity of varying the percentage will be touched upon later.

It is also true that the office attendant may be educated up to administering the "gas", but I have not yet met the one whom I would permit to practice on me or any member of my family. Even despite the closest watchfulness the patient will occasionally lapse into unconsciousness as occurred in one case of Dr. R——'s, where three breaths were sufficient to produce unconsciousness. These lapses into unconsciousness are not without their disagreeable and dangerous features. The patient may have, though rarely, exceedingly unpleasant dreams, the memory of which may remain, or, he may misinterpret something that is said or done during his recovery which may cause a lasting shock to his nervous system or may seriously compromise the dentist, should he be working alone. Should unconsciousness occur, and it is only the trained administrator who can detect it immediately, all work should be stopped, unless an agreement to the contrary exists between the patient and the dentist. It gives me pain which no amount of gas will alleviate to think of the trouble some patients may cause the long-suffering dentist, if he puts them to sleep when he promised to only ease the pain a little. There is something abhorrent to all of us in the thought of being anesthetized and have our lives, as it were, held in the balance by the anesthetist.

I shall not enter here into a discussion of what apparatus is the best. There is no such a thing. Any apparatus which will deliver an even and constant flow of either nitrous oxid or oxygen, as may be desired, will answer the purpose.

As to the technic of administration, the amount of gas or oxygen must be governed wholly and solely by the patient's sensation and, inasmuch as the sense of hearing is the last to go, we can be constantly governed by the patient's answers and signals. Very often the patient will jerk or make reflex movements which may possibly give rise to the impression that pain is being caused. These movements are not always caused by pain, and, on questioning, the patient will declare that he has had no pain. These movements are purely reflex, i. e., they are of spinal origin, and are entirely independent of any perception of pain. These movements very often occur even under deep nitrous oxid anesthesia, the patient being, of course, deeply unconscious.

The amount of nitrous oxid which will be sufficient for the drilling of a sensitive cavity will be totally inadequate for the extraction of a pulp and vice versa; the amount necessary for the extraction of a pulp will cause loss of consciousness if only a sensitive cavity is being prepared. Then again, each individual case will vary as to the amount of nitrous oxid necessary to produce a definite result. We must never lose sight of the fact that it is analgesia and not general anesthesia which is desired.

To summarize:

(1) Nitrous oxid is a valuable and reliable agent for the production of analgesia.

(2) It is absolutely safe.

(3) There are better nerve sedatives than nitrous oxid.

(4) The procedure is expensive and requires some little care in its administration.

In conclusion, I wish to report two rather interesting cases from the practice of Dr. R——. The reports which follow are in the patient's own words and have not been added to in any way by myself.

Case number one being that of a psychologist connected with the University of Cincinnati, is as follows:

"The experience of taking the anesthetic administered to me by Dr. Salzer for the purpose of removing the nerve from the tooth was rather interesting, and not at all unpleasant. The anesthetic takes effect very rapidly. Within a minute or so, I felt a tingling in my fingers. Then I began to lose the feeling of weight, and was scarcely conscious of the chair I was sitting in. At the same time the sensations in the outskirts of consciousness, such as the noises of the street, or voices in the next room, in fact anything except that on which my attention was immediately fixed, became remote and confused.

"Dr. R—— began to work at the tooth as soon as they thought it safe, and Dr. Salzer instructed me to raise my left hand every time I felt the least pain. I had slight twinges of pain when Dr. R—— first began to work, but they were no more severe than those incidental to most operations in dentistry, and I should not have thought of mentioning them if I had not been instructed to indicate every sensation of pain no matter how slight. A few deep breaths of the anesthetic served to lessen the pain still more, and the final operation of taking out the nerve, and going down into the roots of the tooth caused no more discomfort than a twinge of pain about like a sharp needle prick.

"Just before the removal of the nerve, when I was most deeply affected by the anesthetic, I began to shake with laughter. The doctors, I knew, suspected that I had gone over the verge of the consciousness, and was in the hysterical stage of anesthesia, but this was only partially true. I still understood perfectly well all they said, and was able to respond promptly to commands to open my eyes, or clinch my fist. I was also perfectly clear in my own mind as to what I was laughing about, though I doubt if the joke would have seemed so funny without the anesthetic. This was what amused me: I knew that I was near the point of losing consciousness, and nevertheless I could still feel slight twinges of pain. I thought it would be such a joke on the doctors if I reached a point where I could not respond to their instructions to indicate sensations of pain by raising my hand, but could nevertheless still feel the pain. It seems to me now that the joke was decidedly on me, but at the time the idea of the doctors victimizing a helpless patient, who felt pain but could not say so, seemed to me a very absurd outcome to the experiment with the new anesthetic.

"The entire proceeding was over in ten minutes or less, and I am sure I never parted from a nerve in a tooth so easily or so expeditiously before. I suffered a little nausea after the anesthetic, but this was doubtless due to the fact that I forgot Dr. R——'s instructions to eat an early and a very light lunch, and instead ate a late and a very substantial one."

Case number two is that of a physician for whom Dr. R—— prepared four cavities in twenty minutes' time. The following details were jotted down by the patient himself almost immediately after he left the dentist's office:

"After giving my colleague, who kindly administered the gas, an (unsolicited) opinion as to the fit of the mask, I began to smell the gas, which was soon followed by a tingling sensation and I remarked upon an acceleration of the heart beat. This was at once followed by a sense of warmth, which I regarded at the time as vaso-motor relaxation. Then the external stimuli of slight character caused no pain. Gentle application of the bur caused no pain to otherwise very sensitive teeth. More severe application caused a slight sense of pain, which led to reflex muscle movements, indicating my sensation to the anesthetist, because he told me to breathe deeply. I thereby learned at that time, that by increasing the depth of my breathing without being told, I could abolish the realization of pain at will. I was conscious of the dental manipulations, and could readily respond to directions given me. There was at no time any sensation of suffocation or discomfort.

"During the entire process, as far as I know, I was capable of clear reasoning and made mental note of the sensations to report them to the anesthetist. For instance, I was impressed with the clear line of demarcation that separates the realization of outside impressions, such as pain, from reasoning ability. At the time, I thought of a curtain as separating these centers as the best illustration, one or two breaths being sufficient to draw the curtain between the reasoning 'centers' and those which had to do with the perception of pain.

"Inasmuch as the induction of the states of anesthesia and analgesia depend somewhat upon the mental state prior to the administration, I may say, that I was not in the least apprehensive, having taken nitrous oxid on previous occasions to the stage of general anesthesia and having absolute confidence in the ability of the anesthetist.

"While I have emphasized that during the entire time I could reason, there was confusion only once, when the nitrous oxid was discontinued and all the mental processes reestablished themselves in, what seemed to me, 'confusing rapidity.'

"After the analgesia there was no nausea or headache or other ill-feeling, but there was a not altogether pleasing sense of exhilaration, which quieted down within twenty minutes under the influence of a good cigar.

"It is known that auto-suggestion, or suggestion, plays an important role in the effectiveness of an anesthetic; but I may say that prior to the administration I did not believe that in me, the gas could induce such analgesia without anesthesia, and so I stated to my dentist. Therefore, the element of suggestion plays no part in my case."—*Dental Summary, June, 1914.*

---

### GAS-OXYGEN ANESTHESIA. INDICATIONS FOR ITS USE.

BY A. H. MILLER, M. D., PROVIDENCE.

There are a number of surgeons who hold that nitrous oxid and oxygen is the ideal anesthetic, and that it should be

